# Serine-arginine protein kinase 1 (SRPK1), a determinant of angiogenesis, is upregulated in prostate cancer and correlates with disease stage and invasion

**DOI:** 10.1136/jclinpath-2015-203125

**Published:** 2015-10-23

**Authors:** Nicholas Bullock, Jonathan Potts, Andrew J Simpkin, Anthony Koupparis, Steve J Harper, Jon Oxley, Sebastian Oltean

**Affiliations:** 1 School of Physiology and Pharmacology, University of Bristol, Bristol, UK; 2 Department of Urology, Southmead Hospital, Bristol, UK; 3 School of Social and Community Medicine, University of Bristol, Bristol, UK; 4 Department of Cellular Pathology, Southmead Hospital, Bristol, UK

**Keywords:** PROSTATE, CANCER, ANGIOGENESIS

## Abstract

Vascular endothelial growth factor (VEGF) undergoes alternative splicing to produce both proangiogenic and antiangiogenic isoforms. Preferential splicing of proangiogenic VEGF is determined by serine-arginine protein kinase 1 (SRPK1), which is upregulated in a number of cancers. In the present study, we aimed to investigate SRPK1 expression in prostate cancer (PCa) and its association with cancer progression. SRPK1 expression was assessed using immunohistochemistry of PCa tissue extracted from radical prostatectomy specimens of 110 patients. SRPK1 expression was significantly higher in tumour compared with benign tissue (p<0.00001) and correlated with higher pT stage (p=0.004), extracapsular extension (p=0.003) and extracapsular perineural invasion (p=0.008). Interestingly, the expression did not correlate with Gleason grade (p=0.21), suggesting that SRPK1 facilitates the development of a tumour microenvironment that favours growth and invasion (possibly through stimulating angiogenesis) while having little bearing on the morphology or function of the tumour cells themselves.

## Introduction

Prostate cancer (PCa) is the most frequently diagnosed cancer in men in the Western world.^1^ While in the majority of cases the evolution is mild, an important number of patients will eventually progress to the metastatic, castration-resistant form, resulting in PCa also being the second cause of death by cancer in men. The most commonly used classifications are the clinical TNM (tumour, node and metastases) staging (based on the presence of cancer in the prostate, degree of invasiveness in surrounding tissues, involvement of lymph nodes and the presence of distant metastases)^2^ and the histopathological Gleason score (based mainly on the degree of differentiation upon microscopical examination).^3^ The most commonly used serum biomarker for cancer progression is the prostate-specific antigen (PSA). In spite of all these tools being available, there is still an immense challenge to better predict prognosis, distinguish aggressive from indolent forms and therefore guide therapy. To be able to improve management of PCa, we need a better understanding of the biology of this disease.

Angiogenesis is fundamental to tumour growth and has long been recognised as one of the hallmarks of cancer.^4^ Although this process is known to be important in the pathogenesis of PCa, trials investigating the use of anti-angiogenic therapies in PCa have thus far proved disappointing.^5^

Vascular endothelial growth factor (VEGF) is one of the main inducers of angiogenesis.^6^ Over a decade ago it was discovered that VEGF pre-mRNA undergoes alternative splicing to produce both proangiogenic and antiangiogenic families of isoforms, of which only the former is upregulated in PCa.^7^ ^8^ Our group recently described an important VEGF splicing pathway involving serine/arginine-rich splicing factor 1 (SRSF1) and its upstream regulator serine-arginine protein kinase 1 (SRPK1).^9^ ^10^ In addition to identifying SRPK1 activity to be responsible for selective upregulation of proangiogenic VEGF in PCa, we furthermore described the therapeutic potential of small-molecule inhibitors of SRPK1.^9^

SRPK1 is increased in several tumour types including pancreas, colon and breast, with high levels of expression correlating with higher disease grade.^11^ However, the expression of SRPK1 in human PCa has not yet been evaluated on a large scale. The present study therefore aimed to assess SRPK1 expression in human PCa and correlate this with clinicopathological parameters. We also sought to investigate its potential value as a biomarker for the prediction of recurrence after radical prostatectomy.

## Materials and methods

### Patients and ethics

A cohort of 110 patients that underwent radical prostatectomy for PCa at our centre during 2010/2011 were selected from the departmental electronic database on the basis of available pathological specimens and full clinical follow-up. All necessary clinical and pathological details were extracted from the local and/or regional electronic patient record system. PSA follow-up was harvested by a separate investigator who was blinded to all other data. Biochemical recurrence was defined as a PSA level of ≥0.2 ng/mL, with a subsequent confirmatory level of >0.2 ng/mL in accordance with the American Urological Association Prostate Guidelines for Localised Prostate Cancer Update Panel report and recommendations for a standard in the reporting of surgical outcomes. Ethical approval was granted by the National Health Service National Research Ethics Service Committee South West, and all data were anonymised throughout, with investigators blinded to either clinicopathological or experimental data as indicated by their involvement in the study.

### Tissue sampling

Directly after surgery all radical prostatectomy specimens were fixed in formaldehyde and transported to the histopathology laboratory. The outer surface was stained with dye and subsequently cut into transverse sections and embedded in paraffin to allow optimal evaluation of surgical margins. H&E slides were evaluated by a consultant histopathologist with expertise in urological pathology, and an area of representative tumour identified and marked. At the time of the study the section containing representative tumour was recalled from the pathology archives. The tumour was then sampled using a punch biopsy technique, along with an area of normal benign prostate glandular tissue, and re-embedded in paraffin. H&E slides of the selected tissue were then evaluated by a consultant histopathologist with expertise in urological pathology to ensure that the Gleason grade of tumour to undergo immunohistochemistry was representative of that of the total specimen.

### Immunohistochemistry

Immunohistochemistry was performed on 5 μm tissue sections with a rabbit polyclonal primary antibody against SRPK1 (1:200, HPA016431, Sigma-Aldrich) using the Leica BOND III fully automated IHC system (Leica Biosystems). Standard manufacturer protocols were followed after a 20 min period of heat-mediated antigen retrieval. The primary antibody was replaced with standard diluent (Bond Primary Antibody Diluent, Leica Biosystems) for a set number of samples in each batch of staining as a negative control. Human vermiform appendix was used as a positive control for SRPK1.

Staining intensity was scored as negative (0; no staining), weak (1; only visible at high magnification), moderate (2; visible at low magnification) and strong (3; clearly visible at low magnification) by two independent assessors, both of whom were blinded to clinicopathological data. One of the assessors was a consultant histopathologist with expertise in urological pathology. In cases where intensity score differed between assessors, the sample was re-evaluated and a consensus score allocated. More specifically, discrepancy in scoring was encountered in 24.4% of the samples, almost exclusively within one intensity point of one another. At the time of re-review each slide was re-examined in detail by both assessors simultaneously and a consensus score allocated. Both assessors had equal weighting in these discussions.

### Statistical analysis

Differences in SRPK1 expression between benign and PCa tissue were assessed using the Mann–Whitney rank-sum test. All clinicopathological correlations with SRPK1 expression were evaluated using Spearman's rank correlation coefficient since SRPK1 expression is an ordinal variable. Multivariable logistical regression was used to investigate whether clinicopathological variables were associated with increased risk of biochemical recurrence.

## Results

Clinicopathological characteristics of the patient cohort are given in table 1.

**Table 1:** Clinicopathological details of the patient cohort

Variable	Mean (median; range) or n (%)
Age at surgery (years)	64.8 (65.1; 43.7–76.7)
Preoperative PSA (ng/mL)	9.5 (8.8; 1.0–24)
Follow-up time (years)	3.1 (2.8; 0.1–4.1)
Gleason grade	
<7	26 (24%)
7	77 (70%)
3+4	61 (55%)
4+3	16 (15%)
>7	7 (6%)
T-stage	
pT2	60 (55%)
pT3a	40 (36%)
pT3b	10 (9%)
Extracapsular perineural invasion	
Yes	40 (36%)
No	70 (64%)
Extracapsular extension	
Yes	48 (44%)
No	61 (56%)
Positive surgical margins	
Yes	64 (58%)
No	46 (42%)
Biochemical recurrence	
Yes	24 (22%)
No	86 (78%)

PSA, prostate-specific antigen.

Pathological staging of tumours ranged from pT2a-pT3b, with the majority being pT2c (n=51; 46%). Gleason grade ranged from 3+3=6 to 4+5=9, with the highest number of cases being 3+4=7 (n=61; 55%). Mean PSA follow-up was 3.1 years, with 24 patients (22%) experiencing confirmed biochemical recurrence within this period.

**Figure 1:**
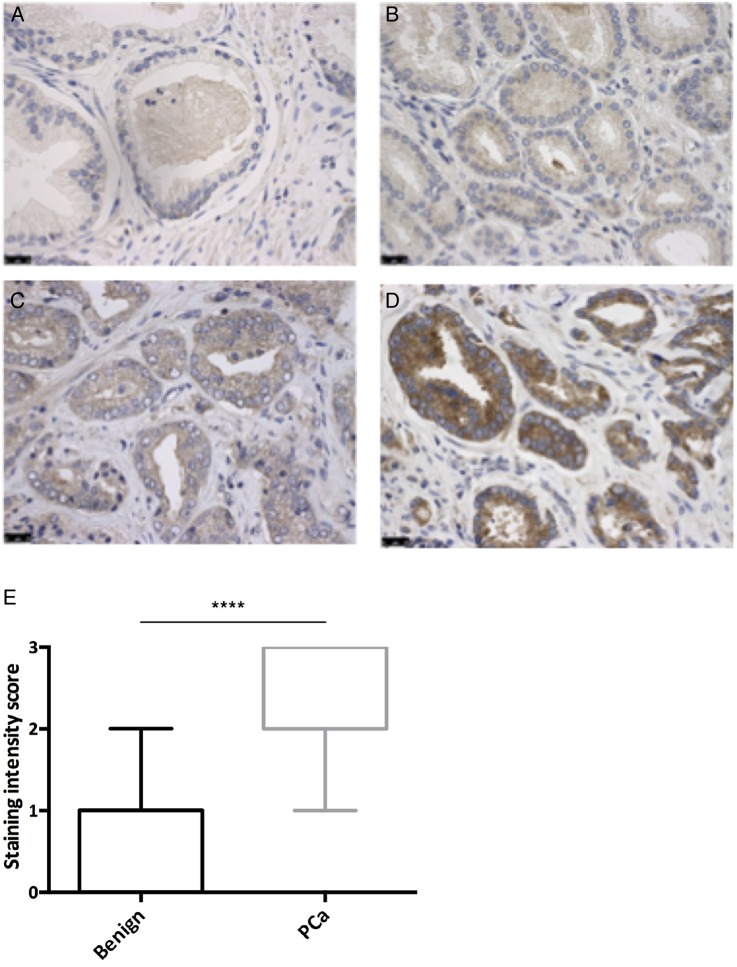
Serine-arginine protein kinase 1 (SRPK1) expression in benign and prostate cancer (PCa) tissue. (A) Benign tissue demonstrating negative SRPK1 staining intensity (score 0). (B) PCa demonstrating weak SRPK1 staining intensity (score 1). (C) PCa demonstrating moderate SRPK1 staining intensity (score 2). (D) PCa demonstrating strong SRPK1 staining intensity (score 3). (E) Box plot comparing SRPK1 staining intensity between benign tissue and PCa. p<0.05 considered statistically significant, calculated using the Mann–Whitney rank-sum test. All images acquired using a light microscope with ×40 objective. Scale bars are 25 μm.

Example images of SRPK1 staining intensity and comparison between benign tissue and PCa are given in figure 1A–D. The expression of SRPK1 was significantly higher in tumour compared with benign tissue, with a median difference in expression score of 2 (IQR 1–3, p<0.00001, Mann–Whitney test; figure 1E).

Correlations between SRPK1 expression and clinicopathological parameters are shown in table 2, along with the results of multivariate logistical regression analysis. SRPK1 expression was strongly correlated with higher pT stage (correlation coefficient 0.27, p=0.004), the presence of extracapsular extension (correlation coefficient 0.28, p=0.003) and extracapsular perineural invasion (correlation coefficient 0.25, p=0.008). SRPK1 expression was not found to be associated with increased risk of biochemical recurrence in our logistical regression model (OR 0.92, 95% CI 0.30 to 2.80), with the only variable identified as having a strong association being Gleason grade (OR 3.47, 95% CI 1.23 to 9.81).

**Table 2:** Clinicopathological correlations with SRPK1 expression and multivariate logistical regression analysis of predictive value for biochemical recurrence

	Correlation with SRPK1 score		Multivariate logistical regression		
Variable	Spearman's rank correlation coefficient	p Value	OR	95% CI	p Value
Age at surgery (years)	−0.02	0.77	1.10	0.98 to 1.23	0.100
Preoperative PSA (ng/mL)	−0.15	0.12	0.97	0.86 to 1.10	0.664
Gleason grade	0.12	0.21	3.47	1.23 to 9.81	0.019
T-stage	0.27	0.004	2.24	0.58 to 8.60	0.239
Extracapsular perineural invasion	0.25	0.008	0.89	0.15 to 5.31	0.902
Extracapsular extension	0.28	0.003	1.07	0.11 to 10.2	0.955
Positive surgical margins	0.08	0.41	2.28	0.67 to 7.75	0.187
Biochemical recurrence	0.08	0.41	NA	NA	NA
SRPK1 expression*	NA	NA	0.92	0.30 to 2.80	0.885

*SRPK1 expression converted to a dichotomous variable for purposes of multivariate logistical regression; high (expression score 3) and low (expression scores 0, 1 and 2).

NA, not applicable; PSA, prostate-specific antigen; SRPK1, serine-arginine protein kinase 1.

## Discussion

This is the first study to demonstrate that SRPK1 expression is significantly increased in human PCa compared with benign tissue, supporting our previously published report that suggests SRPK1 plays an important role in the pathogenesis of the disease.^9^ We found that SRPK1 expression was correlated with higher pT disease stage, extracapsular perineural invasion and extracapsular extension. This may indicate that upregulation of the protein and subsequent promotion of proangiogenic VEGF splicing is an important event in the PCa progression to a more advanced phenotype, thereby adding to the body of evidence supporting SRPK1 as a potential therapeutic target.

**Figure 2:**
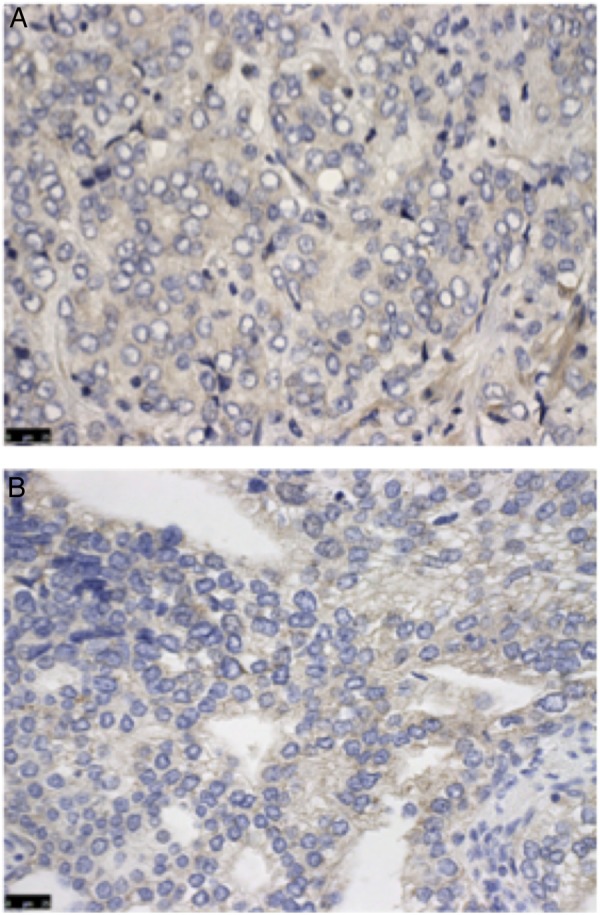
Immunohistochemistry staining of high Gleason grade tumour with low serine-arginine protein kinase 1 (SRPK1) expression. (A) Gleason grade 4 disease demonstrating weak SRPK1 staining intensity (score 1). (B) Gleason grade 4 disease demonstrating moderate SRPK1 staining intensity (score 2). All images taken using a light microscope with ×40 objective. Scale bars are 25 μm.

Studies evaluating SRPK1 in other tumour types identified a positive correlation between expression and grade of disease.^11^ Interestingly, we did not find a significant correlation between SRPK1 expression and Gleason grade in our study cohort. This may suggest that despite being associated with higher disease stage, SRPK1 expression is not necessarily increased in high-grade disease. Indeed, we subjectively observed that those glands exhibiting highest Gleason grade morphology had relatively low SRPK1 expression, as shown in figure 2. One explanation for this difference may be that through promoting proangiogenic VEGF splicing and subsequently boosting new vessel formation in tumours, increased SRPK1 expression facilitates development of a tumour microenvironment that favours growth and invasion while having little bearing on the morphology or function of the tumour cells themselves.

Our logistical regression analysis did not show an association between SRPK1 expression and risk of biochemical recurrence after radical prostatectomy. However, we observed that there was a subjective increase in the percentage of patients experiencing biochemical recurrence as SRPK1 expression increased. This is suggestive of a potential association and indicates that the present study may have been underpowered to detect a predictive effect. Further research using a larger patient cohort is therefore required before asserting that SRPK1 does not have a role in the prognostic stratification of patients following radical prostatectomy.

This study is not without limitations. First, only selected samples of tumour were analysed for SRPK1 expression. It is well known that PCa is often multifocal and heterogeneous and so it is possible that the observed staining intensity of the sample was not representative of other tumour sites within the prostate.^12^ Second, the scoring of immunohistochemistry staining intensity is inherently subjective. We aimed to overcome this issue through scoring being undertaken by two independent assessors, one of whom was a consultant urological histopathologist, using a system that has previously been published in the literature.^9^

**Take home messages**

- Angiogenesis is important in prostate cancer and depends on the presence of the pro- and antiangiogenic VEGF-A splice isoforms.
- SRPK1 is a kinase that drives proangiogenic VEGF-A.
- SRPK1 expression in prostate cancer is increased compared to benign tissue.
- SRPK1 expression correlates with disease stage and invasion.
- SRPK1 expression does not correlate with Gleason score.

## Supplementary Material

Web abstract
