# Supplementary material for: Serine-arginine protein kinase 1 (SRPK1), a determinant of angiogenesis, is upregulated in prostate cancer and correlates with disease stage and invasion
Source: J Clin Pathol. 2015 Oct 23;69(2):171–5. doi: 10.1136/jclinpath-2015-203125 (PMC4752636; doi:10.1136/jclinpath-2015-203125)
Supplement: Web abstract [file jclinpath-2015-203125-s1.pdf]

Factorul vascular de crestere endoteliala (VEGF) este supus la splicing alternativ pentru a produce izoforme atât pro cat și anti-angiogenice. Splicing-ul preferențial a VEGF pro-angiogenic este determinat prin protein kinaza serină-arginină 1 (SRPK1), care este exprimata crescut într-un număr de cancere. În studiul de față ne-am propus sa investigam expresia SRPK1 in cancerul de prostata (CaP) și asocierea sa cu progresia cancerului. Expresia SRPK1 a fost evaluată utilizând imunohistochimia țesutului de CaP extras din probe de prostatectomie radicală de la 110 pacienți. Expresia SRPK1 a fost semnificativ mai mare în comparație cu țesutul tumoral benign ($p < 0.00001$) și corelat cu stagiu pT avansat ($p = 0.004$), extinderea extracapsulara ($p = 0.003$) și invazia perineurală extracapsulara ($p = 0.008$). Interesant expresia nu s-a corelat cu gradul Gleason ($p = 0.21$), ceea ce sugerează că SRPK1 facilitează dezvoltarea unui micromediu tumoral care favorizează creșterea și invazia (probabil prin stimularea angiogenezei) în timp ce are puțin efect pe morfologia sau funcția celulelor tumorale in sine.
